# Exploiting Concurrent Wake-Up Transmissions Using Beat Frequencies

**DOI:** 10.3390/s17081717

**Published:** 2017-07-26

**Authors:** Timo Kumberg, Christian Schindelhauer, Leonhard Reindl

**Affiliations:** 1 Department of Microsystems Engineering – IMTEK, Laboratory for Electrical Instrumentation, University of Freiburg, Georges-Koehler-Allee 106, 79110 Freiburg, Germany; reindl@imtek.de; 2 Department of Computer Science – IIF, Computer Networks and Telematics, University of Freiburg, Georges-Koehler-Allee 51, 79110 Freiburg, Germany; schindel@informatik.uni-freiburg.de

**Keywords:** wireless sensor network, wake-up receiver, concurrent transmissions, constructive interference, wake-up flooding, protocol, beat frequency, MAC protocol

## Abstract

Wake-up receivers are the natural choice for wireless sensor networks because of their ultra-low power consumption and their ability to provide communications on demand. A downside of ultra-low power wake-up receivers is their low sensitivity caused by the passive demodulation of the carrier signal. In this article, we present a novel communication scheme by exploiting purposefully-interfering out-of-tune signals of two or more wireless sensor nodes, which produce the wake-up signal as the beat frequency of superposed carriers. Additionally, we introduce a communication algorithm and a flooding protocol based on this approach. Our experiments show that our approach increases the received signal strength up to 3 dB, improving communication robustness and reliability. Furthermore, we demonstrate the feasibility of our newly-developed protocols by means of an outdoor experiment and an indoor setup consisting of several nodes. The flooding algorithm achieves almost a 100% wake-up rate in less than 20 ms.

## 1. Introduction

Recently, more and more wireless sensor networks [1,2,3,4,5] have been deployed with low-power wake-up receivers. They listen permanently to the wireless channel, but have a marginal and constant power consumption. Only when a wake-up receiver detects a valid wake-up message does it activate the sensor node. Sensor nodes with wake-up receivers are no longer bound to a fixed duty-cycling, but are able to communicate whenever necessary, for example directly following a sensor read-out or when several sensor readings are accumulated into a large transmission package. Consequently, low-power wake-up receivers can greatly reduce the power consumption of wireless sensor nodes, as investigated for example in [6].

**Figure 1:**
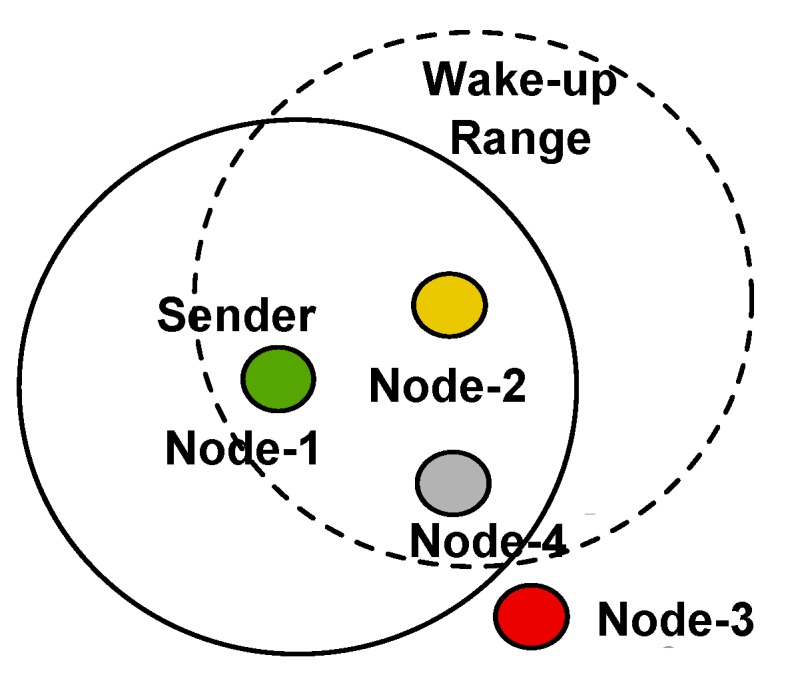
Node-3 is out of the wake-up range due to an outage of Node-4.

However, due to the limitations introduced by the low- to ultra-low-power consumption, the sensitivity of wake-up receivers is usually lower than that of state-of-the-art radio transceivers. In the cases of fading channels, it can happen that a wireless sensor node becomes out-of-reach of any other sensor node, for example due to fading simply caused by changes in the environment or in the network topology that increase path losses. In these cases, a sensor node might become temporarily or permanently disconnected from the wireless sensor network, although it is still alive. Figure 1 shows this scenario where Node-3 is out of the wake-up range of either Node-1 or -2 due to an outage of Node-4.

To improve the reliability and to reduce packet losses, some existing network protocols [7] use concurrent packet transmissions that interfere in a constructive way. The feasibility of concurrent transmissions is demonstrated mainly in the area of IEEE 802.15.4-compliant radios. The IEEE 802.15.4 protocol maps four-bit symbols to 32-bit pseudo-noise sequences that have a length of 0.5 µs at 868 MHz. In order to achieve constructive interferences, this imposes a time constraint of 0.5 µs, which is not easily accomplished.

In this article, we present a novel communication protocol to send wake-up messages by purposefully-interfering signals of two or more wireless sensor nodes, which produce the wake-up signal as the beat frequency of superposed slightly out-of-tune carriers. We investigate the method theoretically and demonstrate its feasibility with the help of several experimental setups. The rest of the article is structured as follows. In Section 2, we briefly introduce the principles of concurrent transmissions, as well as wake-up receivers that use on-off keying-modulated wake-up messages. Furthermore, we introduce the main features of Zippy [8], a flooding protocol that is based on concurrently-transmitted wake-up packets. Then, we investigate concurrent wake-up messages theoretically in Section 3 followed by two newly-introduced communication protocols that are based on concurrent wake-up messages in Section 4. In Section 5, we present the experimental results and conclude our article in Section 6.

## 2. Related Works

In this section, we discuss existing works on wireless transmissions and protocols that deal with radio transmission interference. Secondly, we introduce the basic principles of wake-up receivers and review Zippy [8], a flooding protocol for wake-up receivers.

### 2.1. Wireless Interference

Unintentional interference is often called collision and occurs usually when two or more nodes start to send at the same time and a receiver node detects the signals from both transmitters. However, although the packets collide, it is possible in some cases to correctly receive the strongest signal, which is usually called the capture effect [9,10].

Intentionally-generated interference can lead to constructive interference that can be used to improve link quality or decrease the transmission energy required by a certain node. Usually, protocols that are based on constructive interference can be found in the area of flooding protocols, as they achieve very low latencies due to the concurrently-transmitted packets.

Dutta et al. present in [11] Backcast, a link-layer protocol that relies on identical and concurrently-transmitted acknowledgments as answers to a so-called probe. A probe is sent by an initiator, and each node that receives this probe sends an identical acknowledgment exactly 192 µs after receiving the probe. This means, of course, that acknowledgments from several responders might reach the initiator concurrently. According to Dutta et al., the observed robust detection probability of the acknowledgments cannot be explained only by the capture effect.

One of the first flooding protocols in IEEE 802.15.4-compliant wireless sensor networks that use constructive interference is Glossy [7]. IEEE 802.15.4 uses direct sequence spread spectrum modulation by grouping binary data into four-bit symbols that are mapped to one of 16 possible and nearly orthogonal 32-bit pseudo-noise sequences [12]. The length of one pseudo-noise sequence is 0.5 µs. This also reflects the maximum temporal displacement of two simultaneously-transmitted messages allowed in Glossy to achieve constructive interference [7]. This demonstrates, and Ferrari et al. [7] also conclude, that constructive interference is only possible because of the 802.15.4 modulation scheme and the redundancy generated by the pseudo-noise sequences.

There exist several works that are based on or build upon and analyze the performance of Glossy, such as [13,14,15,16,17,18,19]. These works have all in common that they are based on radios that use the IEEE 802.15.4 standard, and constructive interference of radio packets can be achieved within the synchronization limit of 0.5 µs.

However, a synchronization of 0.5 µs is still very demanding. In Glossy, these temporal constraints are met by synchronizing message transmissions based on signals from the radio to the micro-controller that indicate the reception of a complete packet and by carefully balancing the number of required clock cycles before a transmission begins.

There do not exist many constructive interference-based protocols that are based on other modulation schemes than direct sequence spread spectrum. This is obviously due to the limitations posed by the timing constraints. Zippy, recently presented by Sutton et al. [8], is a flooding protocol that uses wake-up transceivers to synchronize neighboring nodes and to rapidly disseminate data. To ensure the timely waking of all wireless sensor nodes, Zippy transmits concurrent wake-up packets. As these packets are on-off keying-modulated and the wake-up receivers are of low complexity to ensure ultra-low-power consumption, destructive interference has to be avoided, and the capture effect cannot be used. Zippy solves destructive interference by using carrier frequency randomization, as will be further discussed in Section 2.3.

### 2.2. Wake-Up Receiver

**Figure 2:**
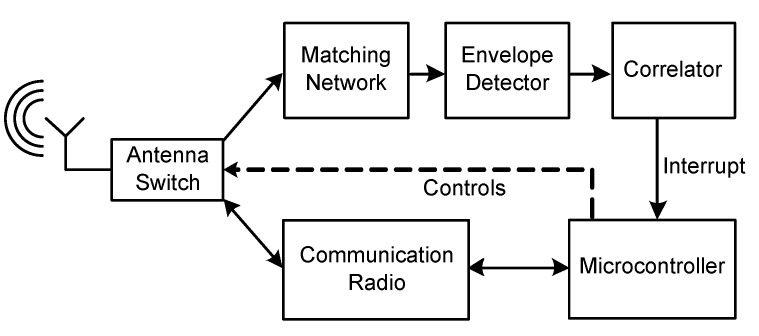
Schematic of the wireless sensor node including a wake-up receiver consisting of the envelope detector and correlator.

Wireless sensor nodes that are equipped with wake-up receivers listen permanently to the wireless channel for incoming wake-up calls. In case they detect a valid wake-up message, they usually provide a signal to the microcontroller of the wireless sensor node that transfers from an energy-saving sleep state to an active state to perform a predefined task. Figure 2 illustrates the main blocks of a wireless sensor node with a wake-up receiver that consists of an envelope detector and a correlator. The matching network provides impedance matching of the antenna and detector. The microcontroller controls an antenna switch and changes, upon reception of a wake-up message, the signal path to a second radio, called the communication or main radio. 

**Figure 3:**
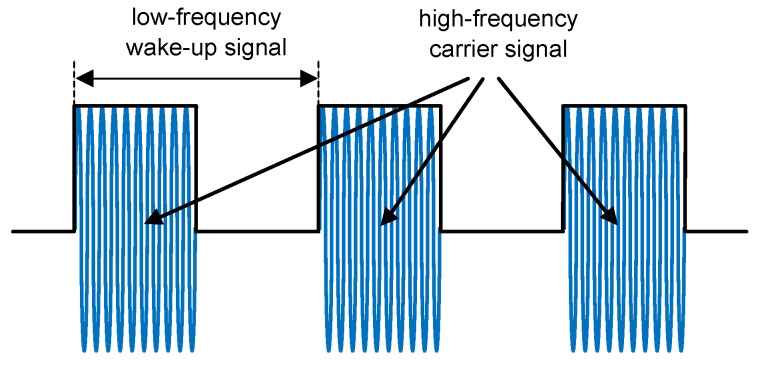
Schematic of a low-frequency wake-up message on-off keying modulated on the high-frequency carrier signal.

The envelope detector demodulates the high-frequency carrier signal to achieve a low-frequency wake-up signal. To ensure ultra-low-power consumption, the demodulator often consists only of passive components like diodes and capacities. Figure 3 illustrates a low-frequency wake-up signal on-off keying modulated on a high-frequency carrier signal.

The above-described wake-up receiver has addressing capability provided by the correlator. The correlator prevents the wake-up receiver from activating the main controller of the sensor node if a wake-up message does not correspond to a pre-configured address. Instead, it resides in ultra-low-power listening state. Examples of wake-up receivers with addressing capability are introduced for example in [2,8,20,21,22,23].

Some wake-up receivers can also be configured to be sensitive to a certain wake-up frequency without the requirement of an address embedded in the wake-up message. Then, the wake-up receiver activates the main controller in all cases that it detects a valid frequency at its input and does not correlate the signal to a stored pattern. An advantage of this method is comprised by short and simple wake-up messages, as they do not require a certain embedded pattern. However, without addressing, wake-up receivers are prone to false positive wake-ups, as shown for example in [8]. Further discussion on wake-up receivers can be found for example in [24,25].

### 2.3. Zippy

**Figure 4:**
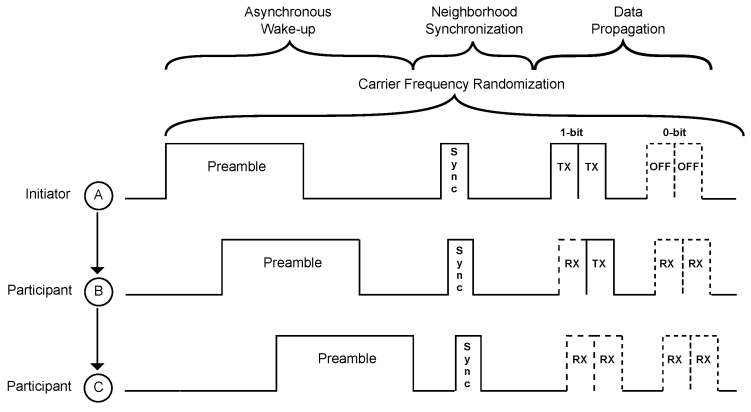
Overview of Zippy.

Zippy, introduced by Sutton et al. [8], is a flooding protocol that uses wireless sensor nodes with wake-up receivers without address correlation, as introduced in Section 2.2. The wake-up receiver tests the frequency of the demodulated signal, and in case it matches 125 kHz, it interrupts the main controller from deep sleep. As illustrated in Figure 4, the node then transmits a wake-up call, here called the preamble, by itself. Figure 4 schematically shows a two-hop Zippy network consisting of the initiator Node A and participating Nodes B and C [8]. As Node A continues to send its wake-up packet after Nodes B and C are awake, all three nodes transmit part of their wake-up packets concurrently to generate a one-hop neighbor time synchronization in the order of milliseconds [8].

After sending the preamble, all nodes go to sleep state for a certain period of time that depends on the number of hops in a multi-hop network. Then, the initiator Node A wakes up again to transmit a synchronization bit (SYNC) as visualized in Figure 4. Node A transmits the SYNC bit in the same matter as the preamble, by using the on-off keying-modulated carrier signal. This SYNC bit then is received by the wake-up receiver of Node B. As the internal automatic gain control unit of Node B’s wake-up receiver has already settled during preamble reception, the detection of the SYNC bit is achieved in the order of microseconds [8]. After receiving the SYNC bit of Node A, Node B itself starts to transmit a SYNC bit. This sequence then continues until no further nodes participate. Using this method, Zippy achieves a mean per-hop synchronization of 34 µs in a two-hop network [8].

After achieving neighborhood synchronization, Node A starts to transmit data using the same on-off keying modulation scheme as before. To ensure fast relaying of the data, the data are transmitted bit-wise and not byte-wise. After Node B receives a bit, it immediately forwards it to Node C.

**Figure 5:**
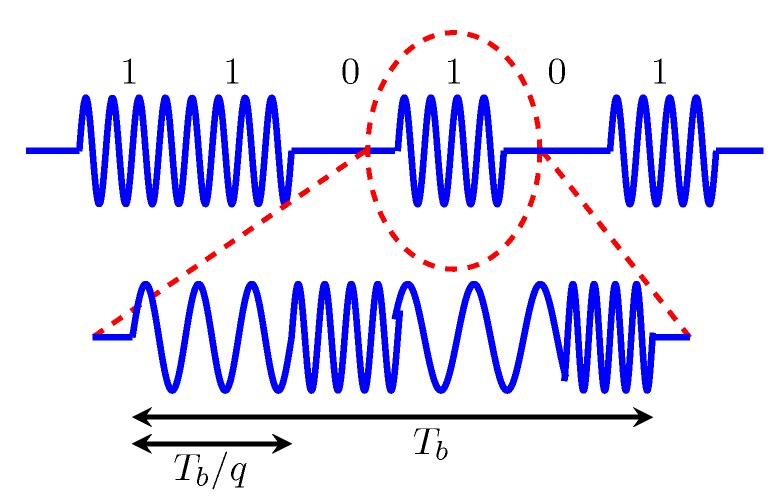
Schematic of an on-off keying-modulated message that implements high-frequency carrier signal frequency randomization.

To mitigate destructive interference during concurrent transmissions, Zippy uses carrier frequency randomization [8]. Figure 5 illustrates an exemplary on-off keying-modulated message that implements carrier frequency randomization. Each low-frequency one-bit consists of several high-frequency periods, which are transmitted at *q* different frequencies. In the example illustrated in Figure 5, each low-frequency bit consists of q=4 frequencies *f* that can be calculated by $f=f_{c}+\beta \Delta$, with $f_{c}$ being the center frequency of 446.8 MHz, Δ= 135.41 kHz resembling a minimum offset between two frequencies and β>=2 being a uniformly-distributed random variable. Using carrier frequency randomization, Sutton et al. experimentally verified a packet reception rate of almost 100% by using six concurrent senders. Using an indoor testbed, Zippy achieves an end-to-end latency between 17.8 ms and 24.4 ms using eight-bit packets and between 29.8 ms and 41.6 ms for 16-bit packets. Here, end-to-end latency is the time elapsed between the start of flooding by the initiator and the end of flooding at each participant [8]. An unsolved challenge of Zippy remains the existence of false positive wake-ups, as Zippy wake-up receivers cannot use address correlation.

## 3. Concurrent Wake-Up Message

As introduced above, one of the major challenges of ultra-low power wake-up receivers is their low sensitivity. Due to this, they are susceptible to signal outages caused by fading channels, and their input signal quickly goes below their sensitivity threshold. As shown in the previous Section 2, constructive interference can improve the reliability of transmissions.

**Figure 6:**
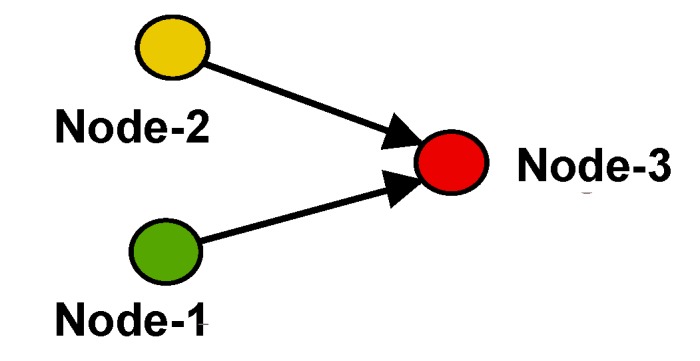
Node-1 and Node-2 send a concurrent wake-up to Node-3.

When two senders transmit the same message simultaneously to a common receiver, constructive or destructive interference can happen. Constructive interference helps the common receiver detect a message and improves the reliability of the transmission [14]. Considering the example given in Figure 6, Node-1 and Node-2 simultaneously send a wake-up message to Node-3 to achieve constructive interference.

### 3.1. Two Concurrent Senders

In case of two concurrently-sent wake-up packets, a possible and probable existing phase-shift ϕ of one of the signals has to be considered. There will be a superposed signal y(t) of the form of Equation (1):

(1) $$y\left(t\right)=A_{1}e^{j\omega_{1}t}+A_{2}e^{j\omega_{1}t}=\left(A_{1}+A_{2}\right)e^{j\omega_{1}t}$$

with $A_{1}$ and $A_{2}\in C$ such that $A_{1}=r_{1}e^{j\phi_{1}}$ and $A_{2}=r_{2}e^{j\phi_{2}}$ with $r_{1},r_{2}\in R$ are the signal amplitudes and $\phi_{2}-\phi_{1}$ the phase shift between the two signals. Furthermore, $\omega_{1}=2\pi f_{1}$ is the angular frequency of the two superposed sinusoidal waves. However, due to the periodicity of the sinusoidal signals, a destructive interference will take place in the range of $2/3\pi <\phi_{2}-\phi_{1}<4/3\pi$, which will finally lead to an unreliable communication.

The superposed signal y(t) as introduced in Equation (1) above is the sum of two sinusoidal signals having the same frequency $f_{1}$. The following Equations (2) and (3) show the same sum of two sinusoidal functions having different frequencies $f_{1}$ and $f_{2}$, but highlight different aspects. Equation (2) expresses y(t) as the sum of two sinusoidal functions with angular frequencies $\omega_{1}=2\pi f_{1}$ and $\omega_{2}=2\pi f_{2}$:

(2) $$y\left(t\right)=A_{1}e^{j\omega_{1}t}+A_{2}e^{j\omega_{2}t}.$$

By replacing $\omega_{2}=\omega_{1}+\left(\omega_{2}-\omega_{1}\right)$, Equation (2) can be rewritten to achieve Equation (3):

(3) $$y\left(t\right)=e^{j\omega_{1}t}\left(A_{1}+A_{2}e^{j(\omega_{2}-\omega_{1})t}\right).$$

**Figure 7:**
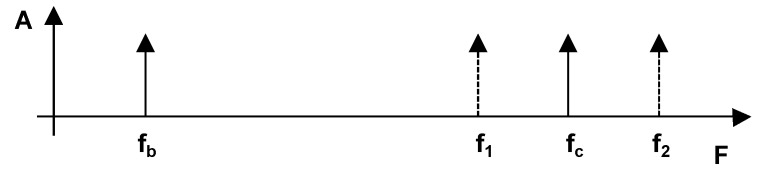
Spectrum of the sum of two sinusoidal functions having frequencies $f_{1}$ and $f_{2}$ and the resulting carrier and beat frequencies $f_{c}$ and $f_{b}$, respectively.

From Equation (3), it can be seen that the amplitudes $A_{1}$ and $A_{2}$ of the original sinusoidal signals are preserved by the addition. By expressing $\omega_{1}$ as $\omega_{1}=\bar{\omega }-\Delta \omega$ and $\omega_{2}$ as $\omega_{2}=\bar{\omega }+\Delta \omega$ and by replacing Δω with $(\omega_{2}-\omega_{1})/2$ and substituting $\bar{\omega }$ with $(\omega_{1}+\omega_{2})/2$, it can be seen that the summation generates two new frequencies that we here call $f_{c}$ and $f_{b}$. The first frequency, $f_{c}$, equals $(f_{1}+f_{2})/2$, and the second frequency, $f_{b}$, equals $(f_{2}-f_{1})/2$. The lower frequency $f_{b}$ is called the beat, as it appears on the left side in Figure 7, which illustrates the spectrum resulting from the sum of frequencies $f_{1}$ and $f_{2}$ of y(t). According to Equation (3), $f_{c}$ is just between $f_{1}$ and $f_{2}$. Equation (3) also shows that a phase-shift ϕ of one of the high-frequency signals is a phase-shift of the beat signal, but no destructive interference between $A_{1}$ and $A_{2}$ takes place. 

With the help of Equation (3), we just demonstrated that a low-frequency signal can be generated by adding two sinusoidal signals with $f_{1}$ and $f_{2}$. Now, assume that a wake-up receiver listens on a certain frequency $f_{w}$, for example 125 kHz, for incoming messages. From the discussion above, it follows that $f_{w}$ can be generated by adding two sinusoidal signals with frequencies $f_{1}$ and $f_{2}$ that are just separated about the right amount from each other. In the example above, with $f_{w}$ = 125 kHz, this would be 125 kHz, since the envelope detector, as long as it consists of two diodes, doubles the frequency during rectification. By switching the $f_{1}$ and $f_{2}$ transmitting radios synchronously on and off, frequency $f_{b}$ can be further on-off keying-modulated to achieve a certain required wake-up pattern. Changing only one of the frequencies $f_{1}$ or $f_{2}$ according to a certain timing, $f_{b}$ can be frequency-shift keying modulated.

Considering Figure 6, Node-1 and Node-2 are usually in an ultra-low-power listening state. To successfully generate a concurrent wake-up message, synchronization of both nodes has to be achieved prior to sending of $f_{1}$ and $f_{2}$. Synchronization can be achieved for example by triggering on the wake-up message that is sent by Node-1 to wake up Node-2 or by utilizing the wake-up acknowledgment transmitted by Node-2. The latter method is implemented in this work, as will be discussed in Section 4.

**Figure 8:**
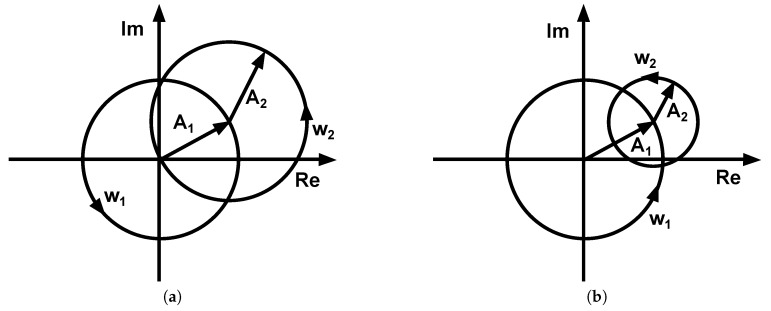
(**a**) $A_{1}=A_{2}$; (**b**) $A_{1}>A_{2}$.

Figure 8 depicts Equation (3) graphically, with the horizontal axis representing the real part and the vertical axis representing the imaginary part of y(t). It can be seen that there exist generally two cases: (a) $|A_{1}\left|=\right|A_{2}|$ and (b) $|A_{1}\left|>\right|A_{2}|$. The case $|A_{1}\left|<\right|A_{2}|$ is the same as Case (b), which may be achieved by reordering $A_{1}$ and $A_{2}$ in Equation (3). Furthermore, it can be deduced from the two graphs (a) and (b) in Figure 8 that the beat signal has an amplitude of $A_{2}$ and $A_{1}$ resembles an offset that shifts the beat signal from the origin. 

**Figure 9:**
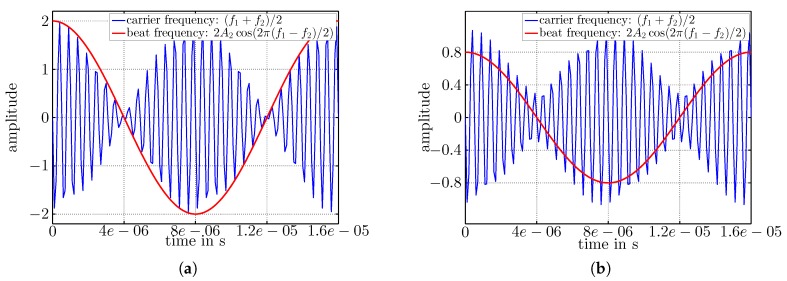
(**a**) Beat for equal amplitudes $A_{1}=1$ and $A_{2}=1$; (**b**) beat for $A_{1}=0.7>A_{2}=0.4$.

Figure 9 visualizes Equation (3) in the time domain; $f_{c}=\left(f_{1}+f_{2}\right)/2$ is shown as the black curve, and $f_{b}$ is visualized in red. In Figure 9a, the two sinusoidal signals have equal amplitudes $A_{1}=A_{2}=1$, and in Figure 9b, the amplitudes are $A_{1}=0.7>A_{2}=0.4$. In both figures, the resulting beat ($f_{b}$) has amplitude $2A_{2}$. The amplitude discrepancy between $f_{b}$ and $f_{c}$ visible in Figure 9b shows the offset generated by $A_{1}$. Please note that the signals plotted in Figure 9a,b were obtained from frequencies $f_{1}=$ 868 MHz and $f_{2}=$ 868.125 MHz, although $f_{c}$ appears to have a lower frequency in the plots, which is due to massive undersampling that was done for reasons of illustration.

**Figure 10:**
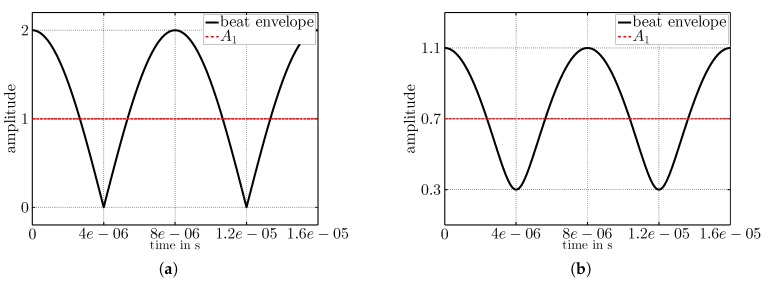
(**a**) Envelope for equal amplitudes $A_{1}=1$ and $A_{2}=1$; (**b**) envelope for $A_{1}=0.7>A_{2}=0.4$.

Figure 10 depicts the same wake-up signal as it appears at the input of the wake-up receiver after envelope detection. As discussed above, the signal has amplitude $2A_{2}$ and frequency $2f_{b}$. Figure 10b also illustrates the DC-offset of $A_{1}-A_{2}$ on the wake-up signal in accordance with the expectations from Equation (3).

As introduced in Section 2.2, wake-up receivers usually listen to the wireless channel in the kilohertz range, with a data rate of several kilobits per second. Consequently, timing constraints are in the µs range. As the wake-up signal appears at the receiver like any other wake-up signal, there are no additional components required, and the same hardware can be used to modulate and demodulate the beat signal.

### 3.2. More than Two Concurrent Senders

In a wireless sensor network, it is probable that more than two senders are in wake-up range, and the superposition of more than two sinusoidal waves can occur. In Section 3.1 above, we discussed that it is possible to transmit a wake-up message from two concurrent senders having frequencies $f_{1}$ and $f_{2}=f_{1}+f_{w}/2$ and amplitudes $A_{1}$ and $A_{2}$. The results obtained in Section 3.1 demonstrate that the amplitude of the larger signal, for example $A_{1}$, generates a constant offset on the wake-up signal. Consequently, if a third node has the same frequency as the node with the lower amplitude, the wake-up signal can be increased, as shown by Equation (4), which enhances Equation (3) by a third sinusoidal signal with amplitude $A_{3}$ and with frequency $f_{2}$:

(4) $$y\left(t\right)=e^{j\omega_{1}t}\left(A_{1}+\left(A_{2}+A_{3}\right)e^{j(\omega_{2}-\omega_{1})t}\right).$$

**Figure 11:**
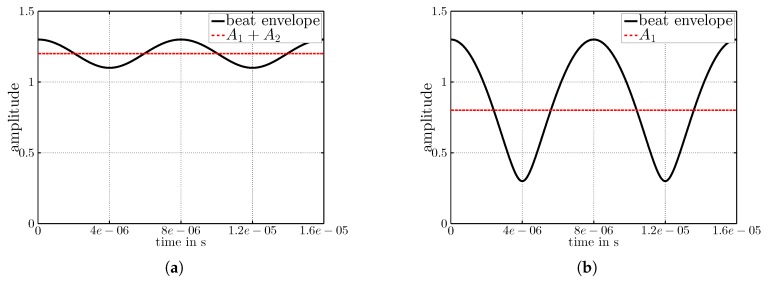
Envelopes for $A_{1}=0.8$, $A_{2}=0.1$ and $A_{3}=0.4$. In (**a**), $f_{1}$ = 868 MHz, $f_{2}$ = 868.125 MHz and $f_{3}$ = 868 MHz; in (**b**), $f_{1}$ = 868 MHz, $f_{2}$ = 868.125 MHz and $f_{3}$ = 868.125 MHz.

The signal y(t) expressed by Equation (4) is graphically reported in Figure 11. Note that the amplitudes in Plots (a) and (b) are $A_{1}=0.8$, $A_{2}=0.1$ and $A_{3}=0.4$. The frequencies in (a) are $f_{1}=868$ MHz, $f_{2}=868.125$ MHz and $f_{3}=868$ MHz. In this case, where $f_{3}=f_{1}$ and $A_{1}+A_{3}>A_{2}$, the amplitude $A_{3}$ of the third node adds to the DC-offset, but not to the beat. In Figure 11b, the frequencies are $f_{1}=868$ MHz, $f_{2}=868.125$ MHz and $f_{3}=868.125$ MHz. In this case, $A_{3}$ adds to the beat and increases its amplitude.

However, due to inaccuracies of the crystal oscillators, frequency $f_{3}$ of the third node will always be slightly different from $f_{2}$ or $f_{1}$. The utilized radio crystals often have an accuracy of 10 to 40 ppm in the kilohertz range, and Equation (4) has to be rewritten. Using $\omega_{1}=\bar{\omega }-\Delta \omega$, $\omega_{2}=\bar{\omega }+\Delta \omega$ and $\omega_{3}=\bar{\omega }+\alpha \Delta \omega$, we get:

(5) $$y\left(t\right)=e^{j\bar{\omega }t}\left(A_{1}e^{-j\Delta \omega t}+A_{2}e^{j\Delta \omega t}+A_{3}e^{j\alpha \Delta \omega t}\right)$$

where α indicates the frequency offset of the third node. Equation (5) indicates that the frequency offset of the third node generates an additional beat (in the lower kilohertz range) that might disturb the wake-up signal. Additionally, the phase of the third node is probably shifted, compared to that of the other nodes, and destructive interference can occur. Due to these limitations, each additional node will likely generate additional frequencies and offsets that make an undisturbed wake-up signal more unlikely, although several wake-up receivers [20,21] have a built-in automatic gain control unit that can help to mitigate this effect.

By expanding Equation (3) by an additional pair of nodes sending at $2\pi f_{3}=\omega_{3}$ and $2\pi f_{4}=\omega_{4}$, we get Equation (6):

(6) $$y\left(t\right)=e^{j\omega_{1}t}\left(A_{1}+e^{j(\omega_{2}-\omega_{1})t}\left(A_{2}+e^{j(\omega_{3}-\omega_{2})t}\left(A_{3}+A_{4}e^{j(\omega_{4}-\omega_{3})t}\right)\right)\right)$$

Considering Equation (6) reveals that four concurrently sending nodes generate the desired superposed beat frequencies in case $f_{2}-f_{1}=f_{4}-f_{3}$. There is also an additional frequency generated by the offset of frequencies $f_{3}-f_{2}$. This offset is present due to oscillator inaccuracies. By setting $f_{3}-f_{2}$ purposefully in the MHz-range, these effects can be reduced, as the additional beats will also be in the MHz range and will be low-passed by the envelope detector. What is left are the inaccuracies of $f_{4}-f_{3}$ not being exactly $f_{2}-f_{1}$.

Equation (6) indicates that *n* nodes can generate n/2 beat wake-up frequencies. However, due to frequency inaccuracies, there will be superposed additional frequencies. Furthermore, mitigating the destructive interferences of the beats needs to be considered, which could be achieved by randomizing the frequencies of node pairs similar to the concept introduced in Zippy [8], although randomizing on the node level, not on the bit level.

**Figure 12:**
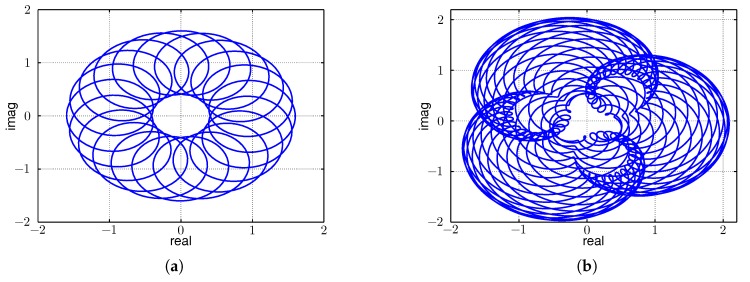
Beats visualized on the real and imaginary axis. (**a**) Two sines at $f_{1}$ and $f_{2}=f_{1}+\Delta f$; (**b**) four sines with $f_{1}$, $f_{2}=f_{1}+\Delta f$, $f_{3}=\alpha f_{1}$ and $f_{4}=f_{3}+\Delta f$.

Figure 12a,b visualizes exemplary beats on the real and imaginary axis. Figure 12a visualizes two sinusoidal signals with frequencies $f_{1}$ and $f_{2}=f_{1}+\Delta f$ and with amplitudes $A_{1}=1$ and $A_{2}=0.6$. It can be seen that the amplitude of the first signal is larger than that of the second, in accordance with Equation (3) and Figure 8. Figure 12b graphically depicts four sinusoidal signals with frequencies $f_{1}$, $f_{2}=f_{1}+\Delta f$, $f_{3}=\alpha f_{1}$ and $f_{4}=f_{3}+\Delta f$ as given in Equation (6). Here, the amplitudes were $A_{1}=1$, $A_{2}=0.6$, $A_{3}=0.3$, $A_{4}=0.2$. The figure demonstrates the additional frequencies as expected from the discussion above.

**Figure 13:**
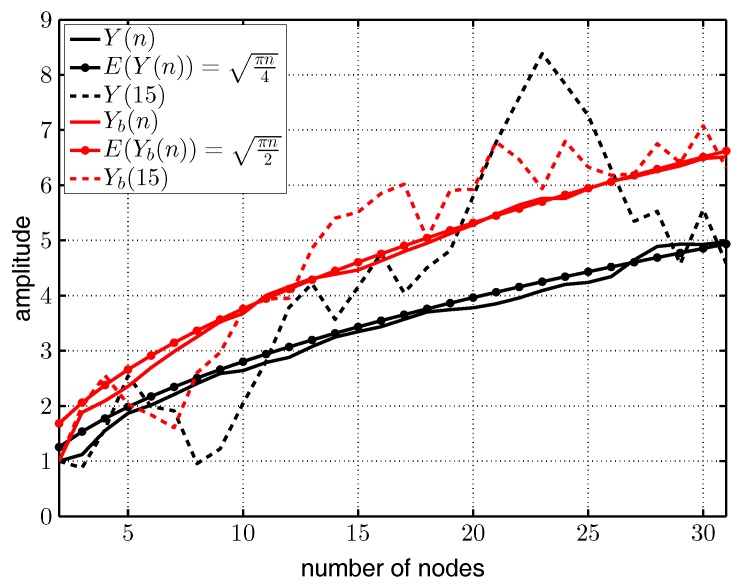
Simulated and expected amplitude achieved by concurrently-sending nodes.

Figure 13 visualizes amplitudes *A* over the number of nodes *n* for concurrently-sending nodes as calculated by MATLAB. The black solid curves are the averaged amplitudes Y(n) for *n* sinusoidal signals (transmitted by *n* nodes) all having the same frequency *f*, a random phase-shift $\phi_{n}$, amplitude A=1 averaged over i=100 runs, as calculated by:

(7) $$Y\left(n\right)=\frac{1}{i}\sum_{1}^{i}\sum_{1}^{n}max\left(A\sin\left(f+\phi_{n}\right)\right)$$

where the function max() extracts the magnitude of the amplitude of each sinusoidal signal. The black dashed curve shows Y(15), exemplary for one sample run. The black dotted curve depicts the expectation value E(Y(n)). Assume the phases ϕ and amplitudes *a* of the sums of *n* sinusoidal signals are normally distributed random variables with μ=0 and $\sigma^{2}=n/2$. Then, $R=\sqrt{\phi^{2}+a^{2}}$ is Rayleigh distributed with $E\left(R\right)=\sigma \sqrt{\pi /2}$. From $\sigma =\sqrt{n/2}$ follows $E\left(Y\left(n\right)\right)=\sqrt{\pi n/4}$.

The red solid curve shows $Y_{b}\left(n\right)$ calculated with Equation (7), but each second signal had frequency f+Δf to generate beat frequencies according to Equation (3). All signals had a random phase-shift $\phi_{n}$ and amplitudes A=1 and were averaged over i=100 runs. The red dashed curve depicts the result of one exemplary run ($Y_{b}\left(15\right)$), and the red dotted curve illustrates the expectation value $E\left(Y_{b}\left(n\right)\right)=\sqrt{\pi n/2}$. The expectation value $E(Y_{b}\left(n\right))$ increased by a factor of two as the variance $\sigma^{2}$ of the beat amplitudes also increased by a factor of two.

Figure 13 does not consider frequency inaccuracies originating from the crystal oscillators, but it demonstrates the advantage of concurrent wake-up signals.

### 3.3. Performance Simulation

**Figure 14:**
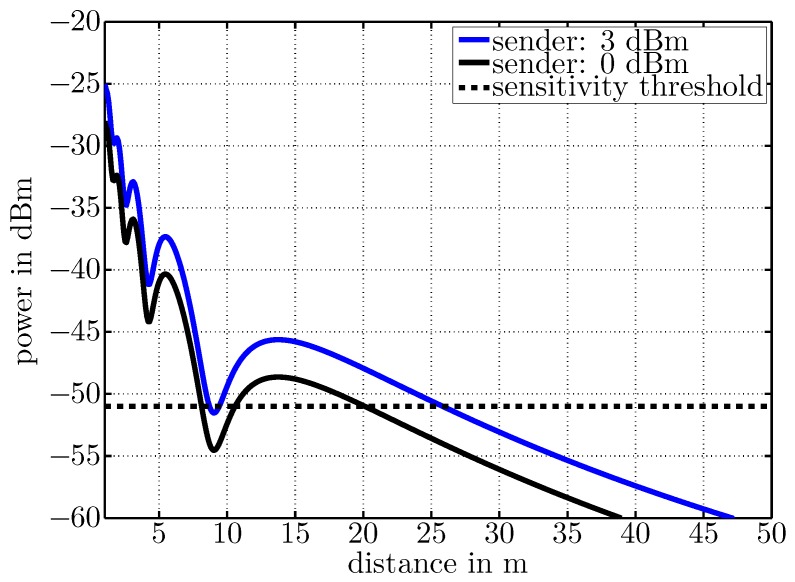
Simulated received signal strength over distance for a transmit power of 0 and 3 dBm.

To evaluate the advantage of concurrent wake-up transmissions of two nearby nodes, we calculated the Friis free-space transmission equation [26] for two different output powers. As the transmission range is additionally affected by multi-path propagation effects like reflection, scattering and diffraction, the simulation included multi-path fading effects to calculate the expected wake-up range [27]. Figure 14 visualizes received signal strength over distance from receiver to sender. During the simulation, the sender and receiver were placed 1.2 m above the ground. The black curve illustrates the expected signal strength for a sending power of 0 dBm and the blue curve for a sending power of 3 dBm. Antenna gain was assumed to be 1.5 dBi in each case. The dashed line shows an exemplary sensitivity threshold of a wake-up receiver at −51 dBm. The curves clearly show the advantages of additional sending power. The wake-up range increases from approximately 20 m to 25 m, and local minima are much less severe. The simulation results will be compared to a free-space measurement in Section 5.3.

## 4. Concurrent Wake-Up Protocol Design

This section presents two prototype network protocols that we developed using concurrent wake-up messages based on beat frequencies. The first subsection introduces a prototype algorithm that can be used to integrate concurrent wake-up messages into existing network protocols, in order to increase their reliability or to decrease the required sending power. The second subsection introduces a prototype wake-up flooding mechanism based on concurrent wake-up messages to achieve a quick and reliable waking up of several sensor nodes.

Throughout this section, we use the abbreviation WUC for a wake-up call and CWUC for a concurrent wake-up call generated by two senders by exploiting the beat frequency.

### 4.1. Integration into Existing Routing Protocols

**Figure 15:**
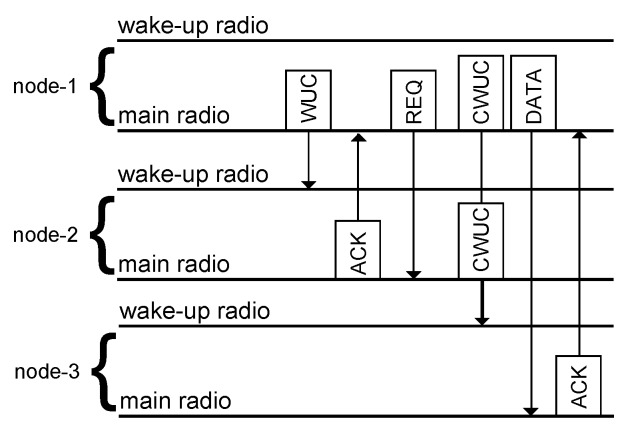
Overview of the concurrent wake-up protocol. Here, we use the abbreviation WUC for a wake-up call, CWUC for a concurrent wake-up call, ACK for the wake-up acknowledgment and REQ for the concurrent transmission request.

Depicted in Figure 15 is a protocol design to demonstrate the feasibility of concurrent wake-up calls and to provide a means to integrate concurrent wake-up calls into existing solutions. To begin a concurrent wake-up, Node-1 sends a single wake-up call (WUC) to Node-2 that acknowledges (ACK) the successful reception. Node-1 then transmits a request (REQ) for a concurrent wake-up message to Node-2 embedding the address of Node-3. Upon reception of this dedicated request, indicated by a special bit in the radio packet, Node-2 changes its carrier frequency for the transmission period of the concurrent wake-up packet. Based on the timing of this handshaking, Nodes-1 and 2 send the concurrent wake-up call (CWUC) to Node-3. After Node-3 detects the valid wake-up call, it is ready to receive data from Node-1. Finally, Node-3 acknowledges the data reception.

In this example, we assumed a scenario similar to that visualized in Figure 1, where Node-3 is out of wake-up range. Alternatively, this scheme could also be used when wake-up messages reach Node-3 with low reliability, only. Both cases could be detected by Node-1, for example, by comparing the number of send wake-up calls to the number of successfully-received wake-up calls. In the case of low wake-up reliability, Node-1 could request Node-2 to transmit a concurrent wake-up message to increase its reliability.

A thorough performance analysis of this approach, including comparisons to existing direct transmission-based approaches, is clearly out of the scope of this work, the goal of which is to demonstrate the feasibility of concurrent wake-up calls based on beat signals. However, we expect beat transmissions to be more effective as the occupied bandwidth is reduced by removing the high data-rate on-off keying modulation. Furthermore, the approach increases the probability of successful wake-up transmissions, which directly leads to a performance gain, especially in fast-fading or noisy environments. This basic concurrent wake-up scheme can be integrated into existing routing protocols, like CTP-WUR (Collection Tree Protocol - Wake-up Receiver) [28], T-ROME [6], ALBA-WUR (Adaptive Load-Balancing Algorithm - Wake-up Receiver) [29] or others, such as [4,30,31].

### 4.2. Concurrent Wake-Up Flooding

Wake-up messages are usually energy-expensive and require a certain amount of time to be sent. In the cases of large wireless sensor node deployments, waking up of all sensor nodes can, as such, pose a considerable amount of energy and time with respect to the wireless sensor network. However, waking up of all nodes is required for example during the initialization phase of a wireless sensor node deployment or more generally required for network configuration tasks and in cases when data need to be pulled out of a complete network. In such cases, it is possible to use flooding algorithms, as they provide a tool to quickly broadcast messages throughout a complete wireless sensor network.

This section introduces a concurrent wake-up flooding protocol similar to Zippy as introduced by Sutton et al. in [8] and as reviewed in Section 2.3. In comparison to Zippy, our newly-developed protocol uses both radios, the main and the wake-up radio. The main radio is used to transmit the on-off keying-modulated wake-up calls (WUC), the concurrently-transmitted wake-up calls (CWUC) and the synchronization (SYNC) messages.

**Figure 16:**
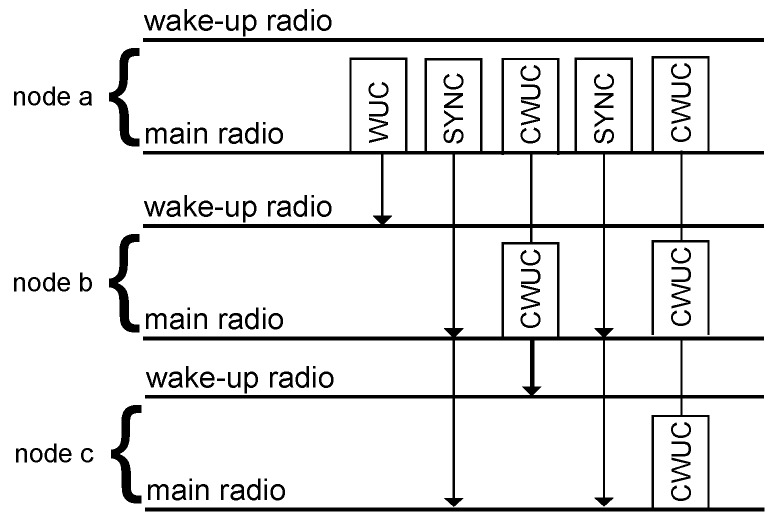
Overview of the concurrent wake-up flooding protocol. Here, we use the abbreviation WUC for a wake-up call, SYNC for the synchronization message sent by the initiator and CWUC for a concurrent wake-up call.

Figure 16 schematically illustrates the concurrent wake-up flooding protocol. An initiator Node a starts the flooding by sending a WUC. Each receiver node of this seed turns its radio on and listens to the wireless channel for a SYNC packet. Based on the timing provided by the SYNC packet, the nodes transmit a CWUC using the beat frequencies. The initiator retransmits the SYNC packet after each CWUC, in order to provide timing information to the newly-awoken nodes. The algorithm finishes at a predefined maximum number of CWUCs.

To the authors’ best knowledge, the herein proposed algorithm is the first concurrent wake-up flooding algorithm that supports address encoding in wake-up packets, which is a clear advantage over Zippy, as it removes virtually all occurrences of false wake-ups [6]. Furthermore, the strength of the beat signal increases due to the concurrency, as shown in the previous Section 3, which leads to a more robust and reliable wake-up reception. Synchronizing on the main radio signals provides improved timing, as well as low latencies, especially in larger network deployments where we expect our proposed algorithm to outperform Zippy.

## 5. Experimental Results

**Figure 17:**
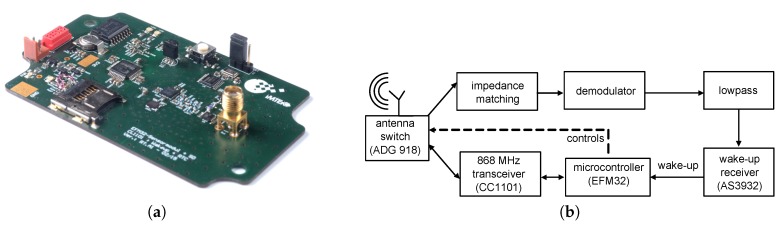
(**a**) Photo of the prototype sensor node used in this work. (**b**) Schematic of the sensor node.

In this section, we present the results of the experiments that we performed to verify the assumptions made above. For the experiments, we utilized wireless sensor nodes as visualized in Figure 17a. Figure 17b illustrates the block diagram of the sensor node schematically. The antenna switch connects in an ultra-low-power listening state the antenna to the low-frequency wake-up receiver AS3932. After detecting a valid wake-up message, the AS3932 awakens the EFM32 microcontroller from the deep sleep state. The microcontroller controls the antenna switch and connects the CC1101 communication transceivers to the antenna to be ready to transmit or receive communication messages.

In combination with the passive demodulator and the 125-kHz wake-up receiver AS3932, the wireless sensor node has a wake-up sensitivity of around −51 dBm. Supplied at 3 V, it has a power consumption of less than 10 µW in ultra-low-power listening state. The AS3932 supports several data rates in the range from 1024 to 8192 bits per second. In the communication mode, the node has a sensitivity of around −100 dBm, depending on the data rate.

### 5.1. Expected Concurrency of Two Senders

**Figure 18:**
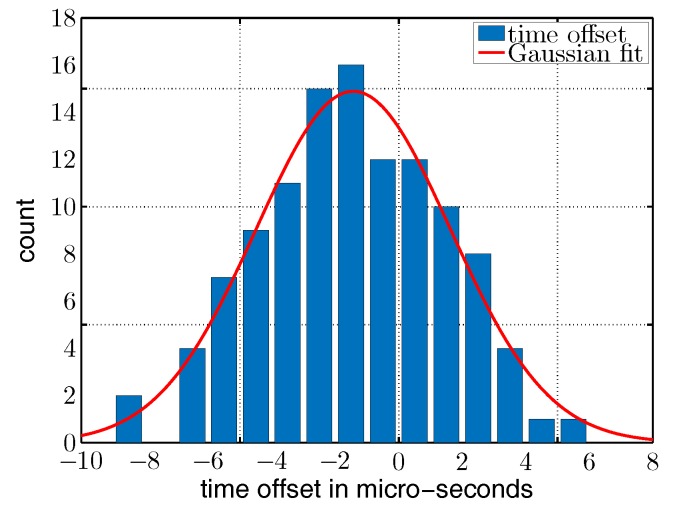
Distribution of time offsets between two concurrent wake-up packets.

To further analyze the concurrency of the wake-up packets, we took the time delays between two concurrent packet transmissions. As the receiver and transmitter have different internal tasks to perform after packet transmission and to prepare to send a wake-up packet, we inserted several *nop()* cycles to adjust the wake-up packets in time. Figure 18 plots the occurrences over delta time $t_{d}$ in µs. We used a Gaussian fit of the form $y=a\exp(-{\left(x-x_{0}\right)}^{2}/\left(2s^{2}\right))$ to evaluate mean $t_{d}=-1.4$ µs and standard deviation s=−3.1 µs. The results of Figure 18 show that this method achieves a timing that is sufficient to send concurrent wake-up packets using the beat-frequency at a data rate of 8192 kbps as required by the wake-up receiver that we used in this work.

### 5.2. Concurrent Wake-up Signals

**Figure 19:**
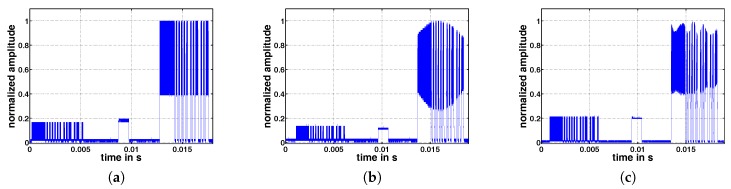
Normalized signal amplitudes received in the case of (**a**) two, (**b**) four and (**c**) six concurrent senders.

Figure 19 shows exemplary concurrent wake-up signals taken at a receiver node. The figures show normalized signal amplitudes over time for the cases of (a) two, (b) four and (c) six concurrent senders. As the signal amplitude is strongly connected to the distance between sender and receiver, the signal amplitudes are normalized to the range from zero to on, in all cases. The single wake-up packet is transmitted in the time period from 0 to 6 ms. After approximately 10 ms, the synchronization packet is transmitted, and the concurrent wake-up packets are transmitted in the time period from 14 to 20 ms. Nodes-1 to -6 sent at an output power of 0 dBm and were placed approximately at a distance of 1 m around the receiver. Nodes-1 and -2 sent with 868 and 868.125 MHz, Nodes-3 and -4 with 866 and 866.125 MHz and Nodes-5 and -6 with 865 and 865.125 MHz. 

It can be seen that in the case of two senders, the signal shape of the concurrent wake-up packet follows the shape as expected in Section 3.1. The beat frequency with amplitude $A_{2}$ is on top of a DC-offset generated by the amplitude $A_{1}-A_{2}$. In the cases of four and six concurrent senders, the signal follows the same shape, but also includes additional low frequencies that are probably caused by offsets of the 125 kHz, as discussed in Section 3.2. However, although the signal amplitudes are not constant over the duration of the wake-up packet, the receiver is able to detect them as valid wake-up messages. To further analyze the impact of additional nodes and to investigate the concept of frequency randomization, further tests are required that will be the subject to our future research.

### 5.3. Free-Space Transmission

**Figure 20:**
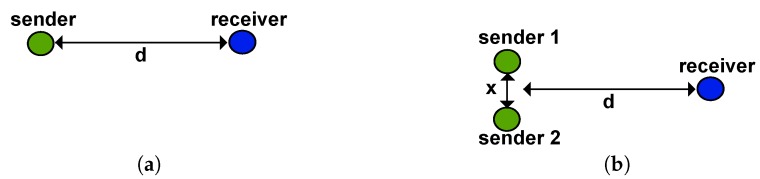
Schematics of the experimental setups for (**a**) single wake-up and (**b**) concurrent wake-up.

To verify the concurrent wake-up, we performed two test setups in free space. The first setup consisted of a single sender and receiver, both at a height of about 1.2 m. The second test consisted of three sensor nodes. Two nodes, placed at a distance *x* = 1 m from each other, sent a concurrent wake-up message. The third node received the concurrent wake-up calls. Both tests consisted of 500 wake-up messages that were sent with a transmit power of 0 dBm. The receiver was placed at different distances *d* in the range from 1 to 50 m and counted each successfully-received wake-up message. Figure 20 shows schematically the experimental setups.

**Figure 21:**
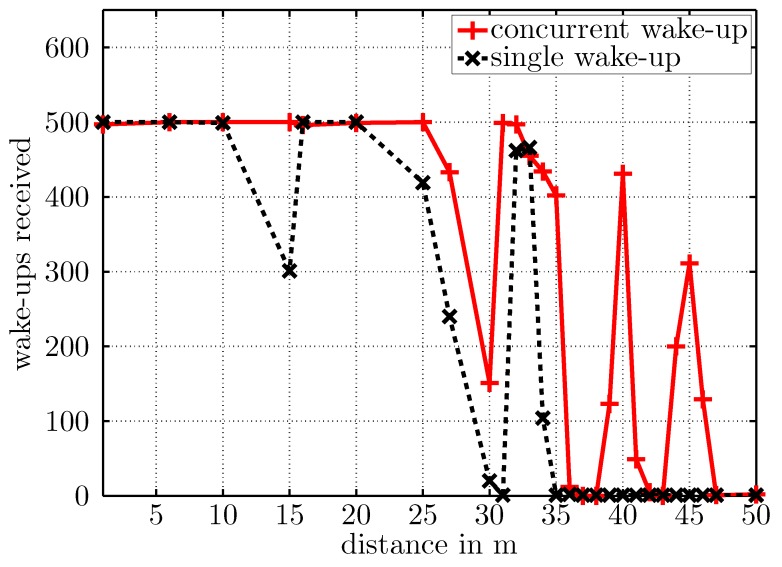
Number of successfully-received wake ups over distance between sender and receiver for single and concurrent wake-ups.

Figure 21 depicts the number of successfully-received wake-up packets over distance. The black dashed curve illustrates the single sender case, and the red curve visualizes the data for the concurrent wake-ups. Both curves correspond well to the simulated free-space transmissions reported in Figure 14, although the local minimum was located at around a 15-m distance for the single wake-up calls and according to the simulation was located at a distance around 8 m. This discrepancy could be caused by a differing ground reflection coefficient from simulation to experiment. The receiver successfully detected each single wake-up message (black curve) until a distance of around 20 m, then the wake-up rate decreased to zero at 30 m. Between 30 and 35 m, the receiver started again to successfully detect wake-up messages that probably originated from additional multipaths generated by surrounding trees or buildings.

The red solid curve in Figure 21 visualizes the amount of successfully-received wake-up messages for the concurrent wake-up calls. The wake-up range increased as expected. Additionally, the minimum at around a 15-m distance to the senders did not exist. The receiver successfully detected each concurrent wake-up call up to a distance of around 25 m. Then, the number of received packets decreased, but not to zero, and increased again at around a 30-m distance. Located at distances of 40 and 45 m were two additional maxima that probably also originated from additional multipath propagations due to scattering effects of surrounding trees, buildings, etc.

### 5.4. Concurrent Wake-Up Protocol

To verify the protocol described in Section 4.1, we enhanced the T-ROME protocol [6] by the concurrent wake-up scheme described in Section 4.1 and implemented it on three wireless nodes to verify its performance. The nodes were placed similarly to the scenario depicted in Figure 1. Node-2 could receive wake-up messages from Node-1, but was not able to receive them from Node-3. Node-2 had data to be sent to Node-3.

**Figure 22:**
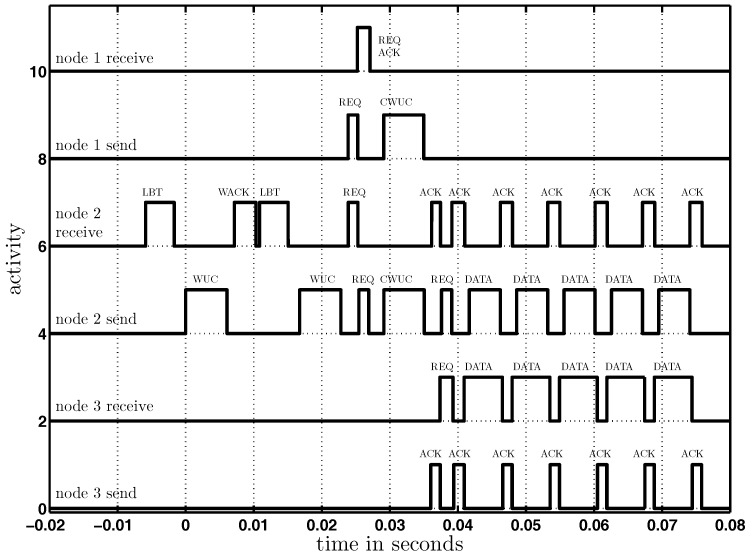
Visualization of the states (send and receive) of Nodes-1, -2 and -3 over time during the described concurrent wake-up protocol. The data were taken with the help of a logic-analyzer. Here, we use the abbreviations LBT for listen-before-talk, WUC for a wake-up call, WACK for the wake-up acknowledgment, REQ for the concurrent transmission request, REQ ACK for the request acknowledgment, CWUC for a concurrent wake-up call and ACK for the data acknowledgment.

Figure 22 visualizes the states (send or receive) of the three nodes. Node-2 started to send a wake-up call (WUC) to Node-3, which did not respond as it was not in wake-up range. Then, Node-2 woke up Node-1 and sent it a concurrent wake-up request, including the address of Node-3. Node-1 acknowledged (ACK) the reception of the concurrent wake-up request (REQ), and based on this acknowledgment, both nodes (1 and 2) sent a concurrent wake-up call to Node-3. Finally, Node-3 verified the wake-up address that was included in the wake-up call and started the handshaking procedure, as introduced in [32], followed by data communication from Node-2 to Node-3.

### 5.5. Concurrent Wake-Up Flooding

**Figure 23:**
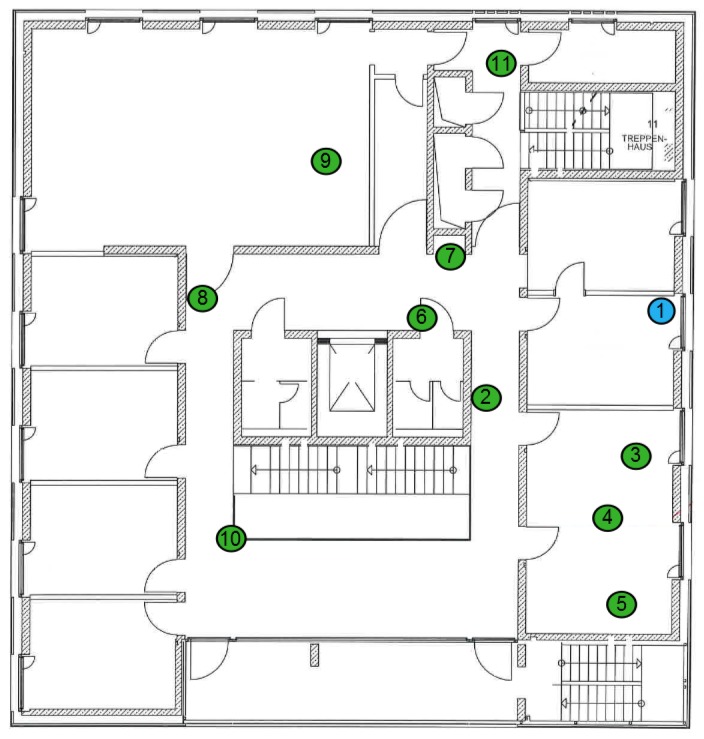
Deployed wireless sensor network to analyze the wake-up flooding algorithm.

**Figure 24:**
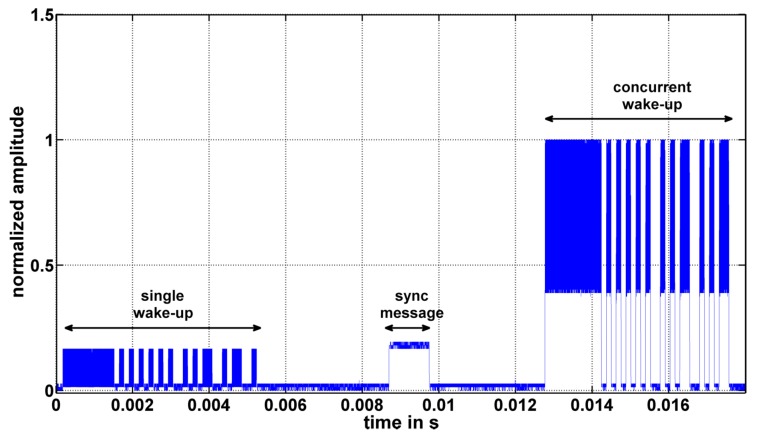
Timing of the wake-up flooding algorithm.

To demonstrate the feasibility of the concurrent wake-up approach utilizing more than two simultaneously-transmitting nodes in a more realistic environment, we implemented the wake-up flooding algorithm as introduced in Section 4.2. We performed an indoor test consisting of several wireless sensor nodes as depicted in Figure 23. The initiator (Node-1), started to broadcast a seed wake-up packet. All nodes (Nodes-2, -3, -4 and -5) that received this seed packet woke up and listened for the synchronization message transmitted directly after the seed packet by the initiator. Based on the timing of the synchronization packet, the nodes then transmitted concurrent wake-up packets on different center frequencies as introduced in Section 3.2. Figure 24 visualizes the timing of the wake-up flooding algorithm. As can be seen, the nodes wake up after around 20 ms and are ready to receive data.

Table 1 lists the frequencies that were utilized by the nodes during the flooding experiment. The third row in Table 1 indicates the type of sensor node during the flooding test, and the last two rows report the amount of sent and received wake-up packets. Types could either be initiator, sender or receiver. The initiator initiated the flooding, and the sender nodes retransmitted the wake-up packets. The receiver nodes were placed in the building at different places where they had no direct link to the initiator, except for Node-5. At each node, we counted the number of successfully received wake-ups.

As reported in Table 1, all nodes had a high wake-up packet reception ratio of almost 100%. As Node-5 could receive all seed wake-up calls, as well as the following concurrent wake-up messages, it woke up 1000 times.

## 6. Conclusions

In this article, we presented a novel communication approach by exploiting purposefully- interfering out-of-tune signals of two or more wireless sensor nodes, which produce the wake-up signal as the beat frequency of superposed carriers. We discussed the theoretical approaches of wake-up messages generated from beat frequencies for the first time and demonstrated their suitability to improve wake-up robustness and reliability. We experimentally verified the theoretical approaches and developed two novel network protocols that apply this technique. First, we developed a simple and highly reliable algorithm that includes concurrent wake-ups into existing protocols. We achieved a quick and reliable dissemination of wake-up messages in a large wireless sensor network, by designing a novel flooding protocol based on concurrent wake-up transmissions. We implemented the algorithms and verified their performance experimentally with the help of an outdoor test setup and an indoor deployment. The tests confirmed the expected gain of up to 3 dB for two concurrent senders and nearly 100% wake-up rates in less than 20 ms achieved by the flooding protocol. Our future research will further investigate the presented protocols and compare their performance to direct transmission-based approaches.

## Figures and Tables

**Table 1:** Frequencies used by the nodes during the flooding test setup.

Node	Frequency in MHz	Type	Number of Wake-Ups Sent	Number of Wake-Ups Received
1	868	initiator	500	–
2	868.125	sender	500	500
3	865	sender	500	500
4	865.125	sender	499	499
5	–	receiver	–	1000
6	866.125	sender	500	500
7	866	sender	483	483
8	–	receiver	–	500
9	–	receiver	–	499
10	–	receiver	–	495
11	–	receiver	–	500
